# Tuning Alginate-Gelatin Bioink Properties by Varying Solvent and Their Impact on Stem Cell Behavior

**DOI:** 10.1038/s41598-018-26407-3

**Published:** 2018-05-22

**Authors:** Zhao Li, Sha Huang, Yufan Liu, Bin Yao, Tian Hu, Haigang Shi, Jiangfan Xie, Xiaobing Fu

**Affiliations:** 1 Institute of Basic Medical Sciences, General Hospital of PLA, Beijing, P. R. China; 2 Key Laboratory of Tissue Repair and Regeneration of PLA, and Beijing Key Research Laboratory of Skin Injury, Repair and Regeneration, First Hospital Affiliated to General Hospital of PLA, Beijing, P. R. China; 3 Medical College, Nankai University, Tianjin, P. R. China; 4 National Research Center of Engineering Plastics, Technical Institute of Physics and Chemistry, Chinese Academy of Sciences, Beijing, P. R. China

## Abstract

Bioink optimization is considered as one of main challenges in cell-laden 3D bioprinting. Alginate-Gelatin (Alg-Gel) hydrogel have been extensively used as bioink. However, its properties could be influenced by various parameters, and little is known about the evidence featuring the impact of solvent. Here we investigated four Alg-Gel bioink by varying solvent ionic strength (named B-1, B-2, B-3 and B-4). Mechanical properties and printability of bioink samples and their impacts on behaviors of encapsulated epidermal stem cells (ESCs) were tested. Bioink with increased ionic strength of solvent showed decreased stiffness and viscosity, and increased swelling and degradation by printability and mechanical property tests. Due to the increased swelling and degradation was associated with shape-maintenance of post-printing constructs, B-3 and B-4 were hardly observable after 14 days. Cellular behaviors were assessed through viability, proliferation, aggregation and differentiation tests. B-2 with optimal properties resulted in higher viability and proliferation of ESCs, and further facilitated cellular aggregation and lineage differentiation. We demonstrated that the solvent can be tuned by ionic strength to control the properties of Alg-Gel bioink and post-printing constructs, which represented a promising avenue for promotion of therapeutic stem cell behaviors in 3D bioprinting.

## Introduction

Three-dimensional (3D) bioprinting shows potential in tissue engineering^1^ and regenerative applications due to its overwhelming advantages over other approaches. Despite advance in bioprinting and biofabrication during the past decade, fabricating complex and functional tissue constructs that mimic their natural counterparts still remains a challenge^2,3^. In order to promote the functions of bioprinted tissues, the development of novel and versatile bioinks will have crucial implications^4^. To fulfill certain physiological and biological needs, the ideal bioink should not only be plastic, printable and suitable for specific 3D bioprinters, but also serve as nontoxic and biocompatible extracellular matrices (ECM) or scaffolds that facilitate the biological behaviors of seed cells^5^. Natural derived materials are famous for the excellent biocompatibility and abundance, among which sodium alginate mixed with gelatin (Alg-Gel hydrogel) has been widely used as bioink for extrusion-based 3D bioprinting^6–10^.

On the other hand, scientists have validated that changing properties of bioink will significantly impact cell behavior and tissue formation within printed constructs^11–13^. Especially, viscosity of bioink in gel phase is crucial for the extrusion-based 3D printing process and stiffness of bioprinted constructs significantly influence embedded cell behaviors^14–16^. Chuang *et al*. demonstrated that Alg-Gel bioink with higher viscosity facilitated higher print resolution and precision in comparison with alginate solution and pre-crosslinked alginate. Meanwhile, viability of primary myoblast in Alg-Gel based bioink was not affected by printing process, which indicated that Alg-Gel hydrogel with proper mechanical property protected embedded cells from shear force when extruded out of printing nozzle^17^. Ouyang *et al*. discovered an optimized formula of Alg-Gel based bioink for embryonic stem cells based on printability and embedded cell behaviors of bioinks with different mechanical properties^18^. Therefore, appropriate tune methods are essential for the comparison of bioinks with different properties and the screen of bioinks for specific application.

Developing the desired tune methods for bioinks is of great importance for the promotion of cell-laden bioprinting. The appropriate tune methods will provide effective means to tune the mechanical and physical properties of bioinks in a controllable manner and most importantly, can facilitate both design and printability of existing bioinks^19^. There is also a concern for safety in the tuning process. As the bioink is required to interact with cells *in vitro* and *in vivo*, PBS was selected as the solvent of the bioink for its cytocompatible. A few studies have reported the impact of solvent on hydrogel^20^, yet have not reported such tunability and responsive cell behaviors. In this work, we presented an easy method of tuning Alg-Gel bioink properties by varying ionic strength of PBS (phosphate buffer saline). We demonstrated 4 formulations to customize bioink by different solvent ionic strength, in order to optimizing properties as well as maintaining fine printability. Additionally, we uncovered the biological performance including viability, proliferation, aggregation and differentiation of stem cells within the corresponding 3D bioprinted constructs.

## Results

### Printability and Mechanical Properties of Bioinks

**Figure 1:**
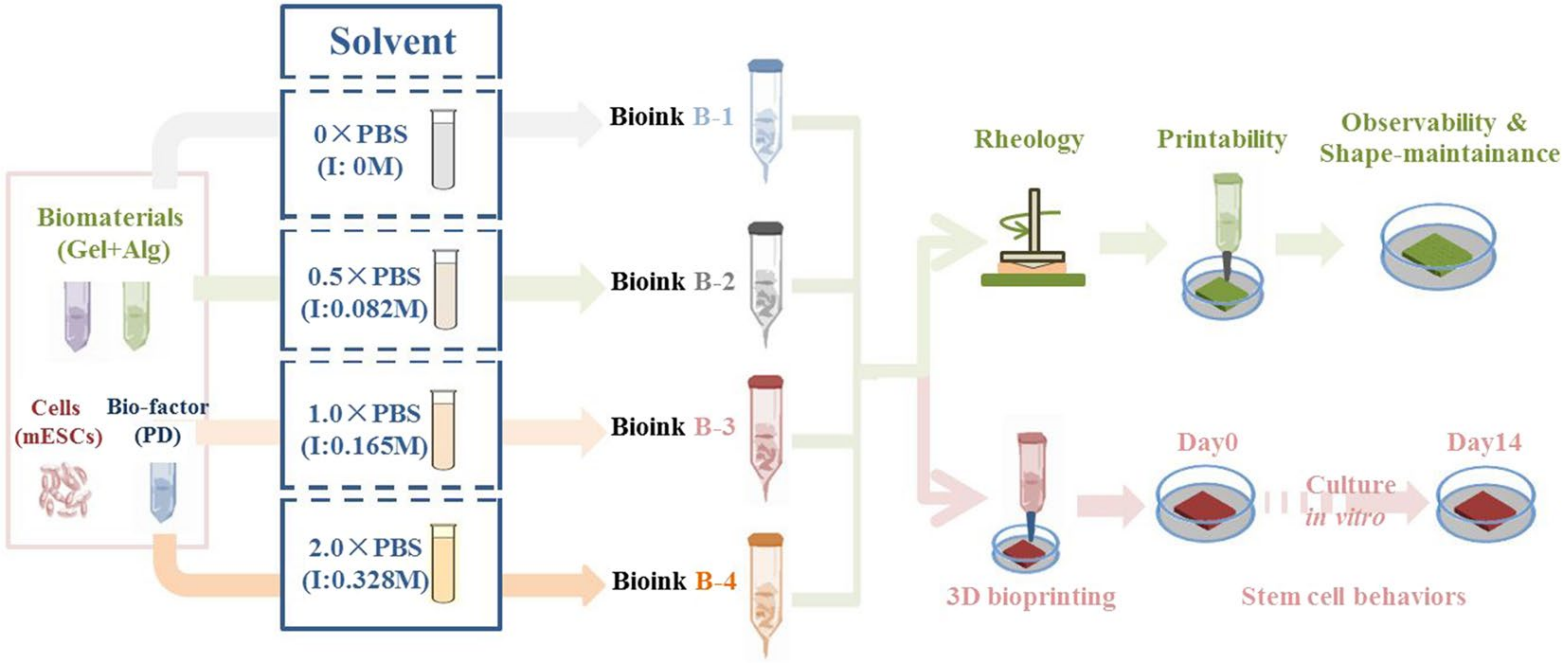
Schematic representation of this research (Gel: gelatin; Alg: alginate sodium; mESCs: mouse epidermal stem cells; PD: homogenate of mouse plantar dermis; PBS: phosphate buffer saline; I: ionic strength).

We hypothesized that the printability and mechanical properties of Alg-Gel hydrogels could be tuned by changing solvent ionic strength, resulting in the regulation of stem cell behaviors within the hydrogels after bioprinting. According to the viability and proliferation of embedded stem cells in different bioinks, the Alg-Gel bioink which promoted cell behavior the best was chosen for further studies of cell differentiation and aggregation (Fig. 1).

**Figure 2:**
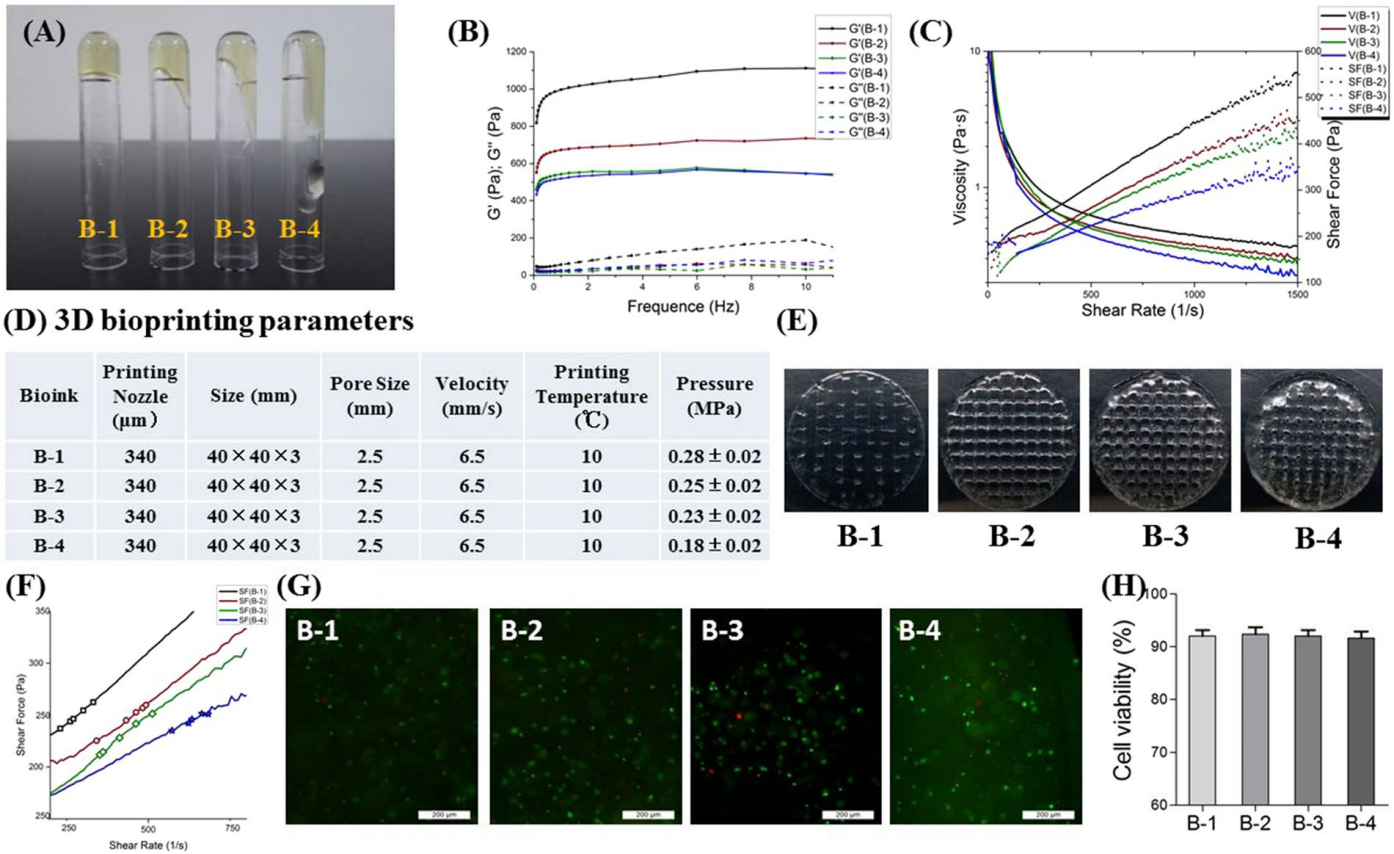
Mechanical properties and printability of bioinks (**A**) Optical images showing the fluidity of pre-cooled bioink after 10 min still standing upside down at 10 °C; (**B**) Storage moduli (G’) and loss moduli (G”) of bioink at 10 °C; (**C**) Viscosity/Shear Force-Shear Rate Curve of bioink at 10 °C; (**D**) 3D bioprinting parameters; (**E**) Printability of bioink represented by actually printed constructs with different line continuity and spreading ratio under the same printing pressure of 0.20 MPa; (**F**) Actual shear force on cells (colorized dots) during bioprinting process (*p* > 0.05); (**G**) Representative images of Live/Dead staining of 3D bioprinted constructs right after bioprinting (Day0) (Scale bar: 200 μm); (**H**) Live/Dead cell counting and quantitative analysis right after bioprinting (*p* > 0.05).

All four Alg-Gel bioinks (named B-1, B-2, B-3 and B-4) exhibited gel-like behavior (Fig. 2A) at operating temperature (10 °C) on printer, as indicated by higher storage moduli (G’) than loss moduli (G”) according to the frequency tests (Fig. 2B). With decreasing of PBS ionic strength, Alg-Gel based bioink showed higher storage moduli (G’) and could better maintain the shape. Bioink with lower solvent ionic strength also showed higher viscosity and shear force between the shear rate of 0.1–1500 sec^−1^ (Fig. 2C), which indicated that bioink with better shape-maintenance acquired more pressure during extrusion of printing.

According to our printing practice, all four bioinks could be printed successfully once the printing parameters, such as pneumatic pressure were regulated properly (Fig. 2D). Porous cell-laden constructs of four bioinks were fast fabricated according to our printing process. Optically visible crisscross cylinders with pores were created as was shown in Fig. 2E. However, when printed under the same pressure, B-1 with high viscosity, required higher pressure and was prone to fragile, while B-4 showed high spreading ratio.

In order to acquire the specific shear force on cells in bioink when extruded out of printing nozzle, 2 ml Alg-Gel hydrogel of each group was extruded out as continuous line under appropriate pneumatic pressure in printing practice. To achieve continuous extruded line, bioinks in different groups were extruded with different shear rate due to different stiffness and viscosity. Finally, since bioink with higher stiffness was extruded with lower shear rate, cells in high-stiffness bioink suffered nearly the same shear force with in low-stiffness bioink according to the Shear Force-Shear Rate curve (*p* > 0.05) (Fig. 2F), resulting in nearly the same cell viability in all constructs of four groups right after printing without significant difference(*p* > 0.05) (Fig. 2G,H).

### Characteristics of 3D Constructs Post-Printing

**Figure 3:**
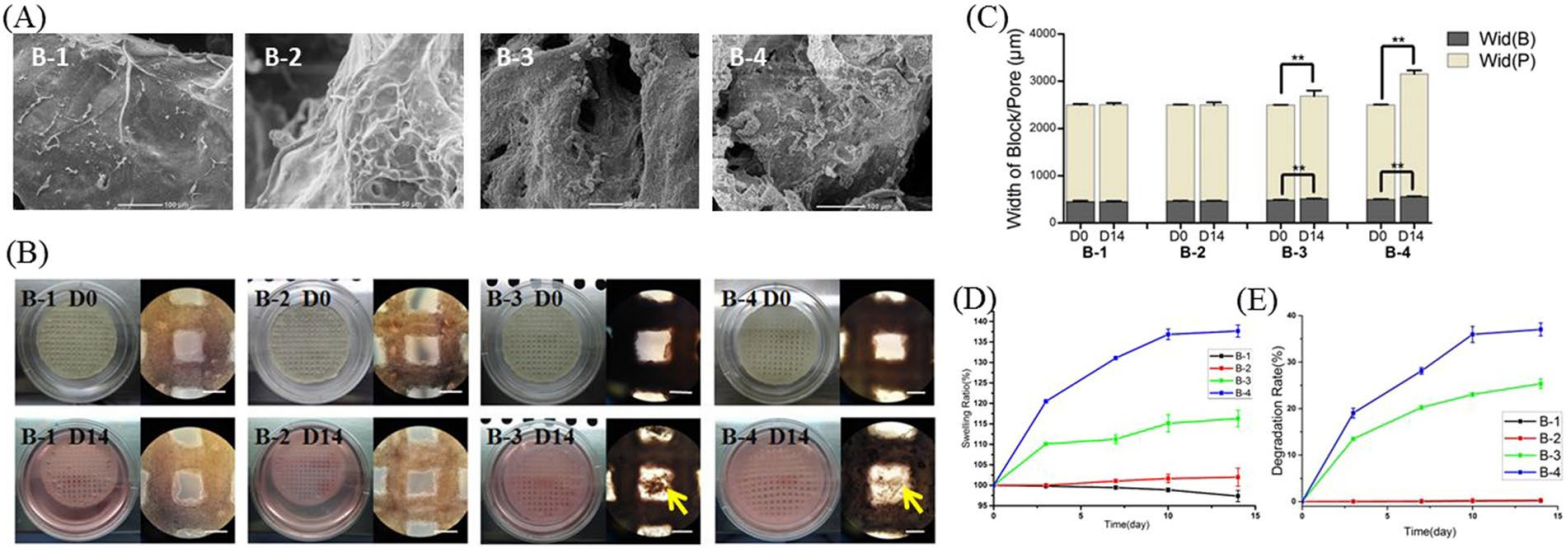
Characteristics of bioprinted constructs (**A**) SEM images of 3D bioprinted constructs; (**B**) Optical and light-microscope images at Day0 and Day14 (scale bar in light-microscope images: 500 μm, yellow arrows showed the flocculent precipitate scattered in pores); (**C**) Quantitative analysis of Width of cylinder (**B**) and pore (P)(^**^p < 0.01); (**D**) Swelling ratio (expended volume of cylinder/initial volume of cylinder) of bioprinted constructs; (**E**) Degradation rate (reduced weight/initial weight) of bioprinted constructs.

The bioprinted constructs showed different surface property (Fig. 3A). B-1 was characterized by flat surface, while B-2 was characterized by rugged ravine with more sufficient cell spreading and connections. With the increase of solvent concentration (B-3 and B-4), the surface of bioink became loose and granular in which cells were clustered for being wrapped by Alg-Gel hydrogel, and less spreading.

Under light microscope, B-1 and B-2 were transparent with uniform distribution of cells while B-3 and B-4 were non-transparent with fuzzy images of cells (Fig. 3B). There was no significant difference among the diameter of constructs and the width of cylinders (*p* > 0.05) and pores (*p* > 0.05) in all four groups right after printing (Fig. 3C). After 14 days of culture *in vitro*, B-1 and B-2 remained transparent and no significant swelling and spreading of diameter (*p* > 0.05), cylinders (*p* > 0.05) and pores (*p* > 0.05) were discovered in these constructs. However, constructs of B-1 and B-2 showed optically swelling with significant spreading of diameter (*p* < 0.01) and width of cylinders and pores (*p* < 0.01), compared with those at Day0 (Fig. 3D). Meanwhile, flocculent precipitate scattered in pores was a sign of apparent degradation in B-3 and B-4 at Day14 of culture (Fig. 3E).

### Cell Viability and Proliferation within 3D Constructs

**Figure 4:**
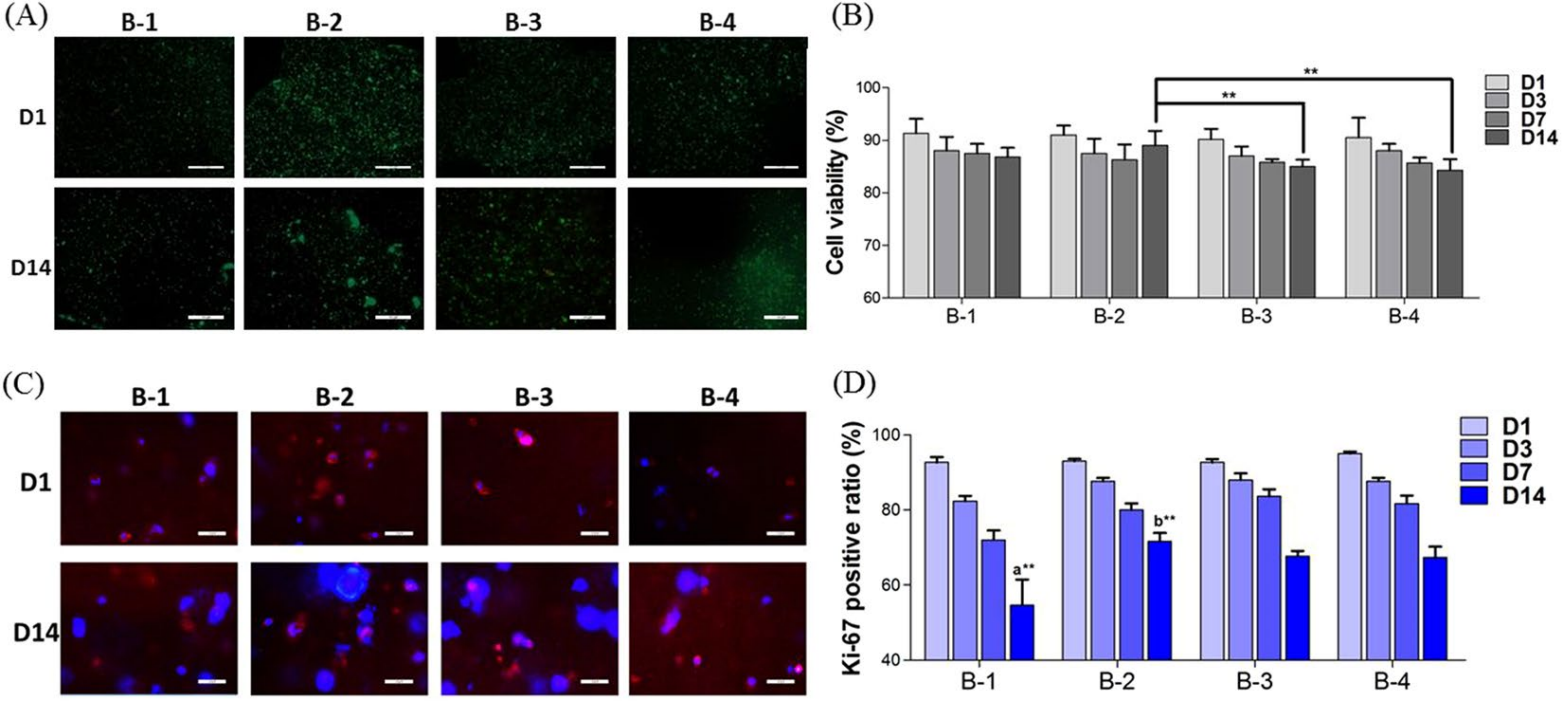
Cell viability and proliferation within 3D constructs (**A**) Representative images of Live/Dead staining of 3D bioprinted constructs at Day1 and Day14 of culture (Scale bar: 200 μm); (**B**) Live/Dead cell counting and quantitative analysis at Day1, 3, 7 and 14 of culture(^**^p < 0.01); (**C**) Representative images of Ki-67 staining of 3D bioprinted constructs at Day1 and Day14 of culture (Scale bar: 50 μm); (**D**) Ki-67 positive cell counting and quantitative analysis at Day1, 3, 7 and 14 of culture (a: ^**^p < 0.01 compared with B-2, B-3 and B-4 at Day14; b: ^**^p < 0.01 compared with B-3 and B-4 at Day14).

Minimizing the pneumatic pressure and speed of printing nozzle achieved higher cell viability (>80%) in our experiment (Fig. 4A). A decreasing trend of cell viability during first 7 days of culture was detected by Live/Dead staining in all four groups (Fig. 4B). However, a modest rebound during Day7 and Day14 of B-2 was significantly different compared with B-3 (*p* < 0.01) and B-4 (*p* < 0.01) in the same period.

A decreasing trend of cell proliferation was detected by Ki-67 staining during 14 days of culture in all four groups (Fig. 4C). Faster decline of cell proliferation was detected in B-1, in which Ki-67 positive rate was under 50% at Day14 (Fig. 4D). However, cell proliferation in B-2 was the highest at Day14 compared with other constructs (*p* < 0.01).

### Cell Aggregation within 3D Constructs

**Figure 5:**
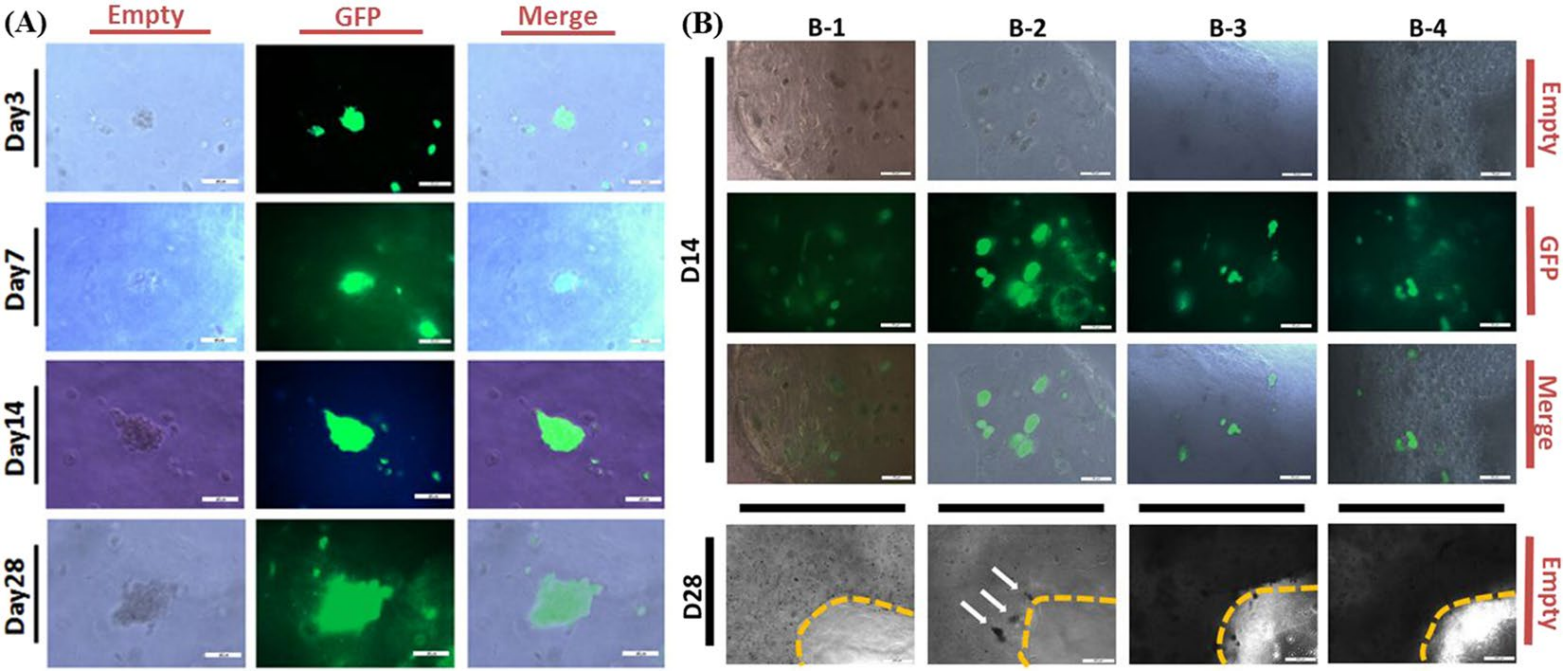
Images of cell aggregation within 3D constructs (**A**) Images of glandular cell clusters discovered in the B-2 during 28 days of culture (Scale bar: 50 μm); (**B**) ESC clusters formation in constructs at Day14 (Scale bar: 50 μm) and Day28 (Scale bar: 200 μm) of culture (White arrows: ESC clusters).

The cell aggregation in clusters was often recognized as a primitive sign of sweat gland morphogenesis. Plenty of cell clusters were observed in B-2 from Day14 to Day28 of culture, indicating that cell clusters could be formed with a glandular morphology inside the bioprinted constructs (Fig. 5A). While large amount of cells were still individual in the cylinders of B-1, B-3 and B-4 (Fig. 5B), the optimized bioink (B-2) was chosen for the further differentiation analysis.

### Cell Differentiation within 3D Constructs

**Figure 6:**
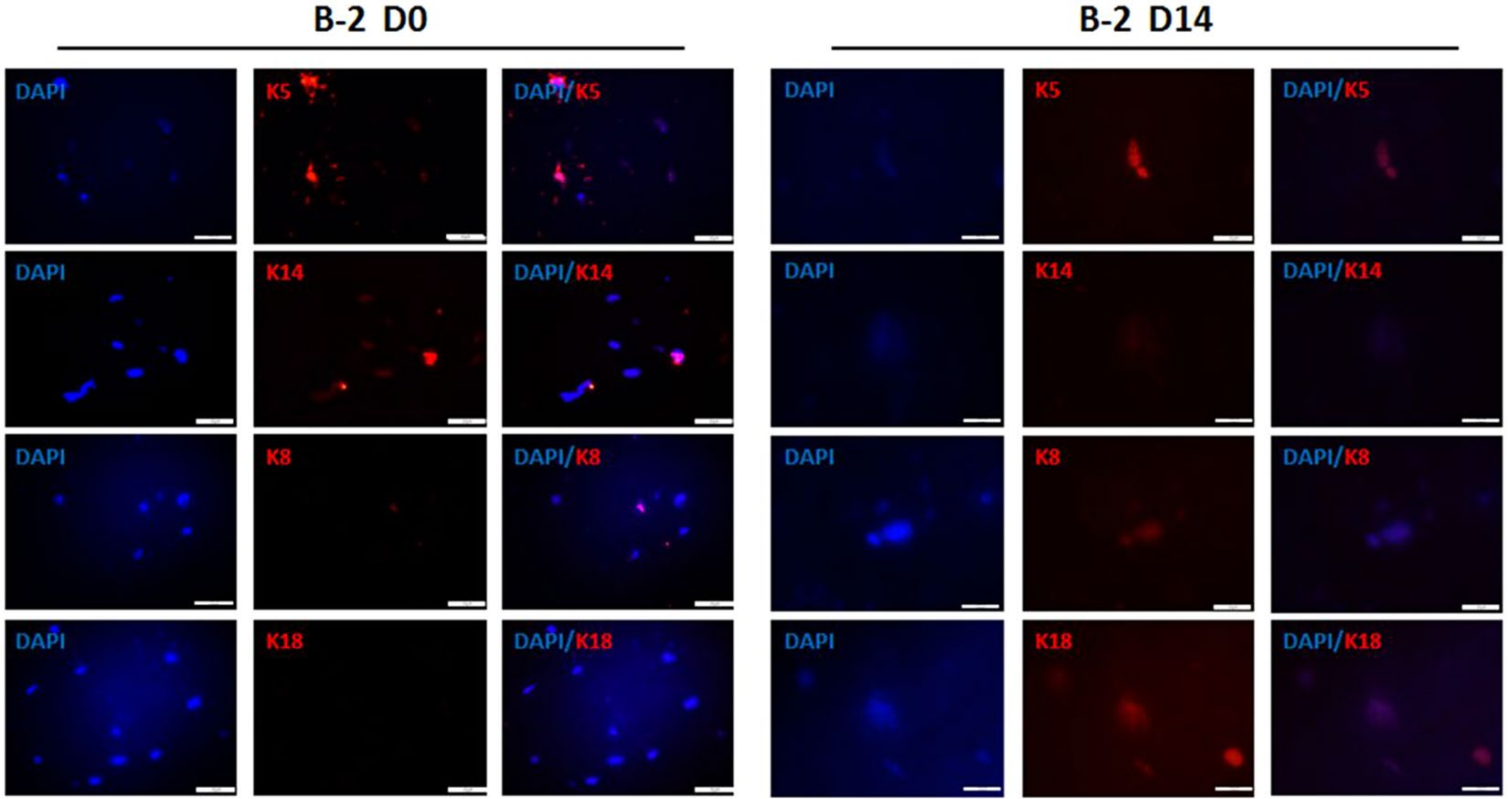
Immunostaining assay with K5, K14 for detection of stemness and K8 and K18 for sweat gland differentiation of epidermal stem cells at Day0 and Day14 of culture in B-2 (Scale bar: 50 μm).

To test whether these cell clusters were glandular-lineage differentiated, typical indicators of epithelium (K5, K14) and sweat glands (K8, K18) were investigated (Fig. 6). At Day0, cells embedded in B-2 exhibited primarily epidermal cell phenotype, as indicated by K5 and K14 staining, while cells exhibited high levels of sweat gland differentiation at Day14, as indicated by K8 and K18 staining.

## Discussion

In this work, we demonstrated an approach to modulating bioink properties by varying solvent ionic strength of Alg-Gel hydrogel, and indicated that this modulation had a profound effect on stem cell behaviors in 3D bioprinting. Several recent studies used alternative approaches to examine the role of altered hydrogel properties on cell biology^21–23^, but the approach described in this paper was easier to manipulate in that only solvent of bioink is utilized without modification of ingredients and hydrogel concentration. Moreover, this approach allowed modulation of bioink properties over a range in a controlled manner and maintained printability, providing a homogeneous microenvironment to cells for probing their behaviors. This approach to optimize the initial modulus associated with Alg-Gel bioink properties, might also have implications in advanced development of existing bioinks.

Several researches demonstrated that cell viability was low (40–80%) in extrusion-based bioprinting for the high extruded shear force from printing nozzle on cells^24^. However, higher cell viability (>80%) was achieved in our experiment due to minimizing the pneumatic pressure and velocity of printing nozzle which could be regarded as “practice-oriented” bioprinting and was differed from bioprinting with fixed pneumatic pressure and velocity of printing nozzle. Noticeably, the practice-oriented bioprinting resulted in the same cell viability in all 3D constructs in the first 7 days of culture. Theoretically, cell viability was greatly influenced by the microstress when they were extruded out of printing nozzle. Microstress was determined by the shear force of corresponding bioink. Microstress on cells could be calculated according to the corresponding shear rate. Since there was no significant difference of actual microstress on cells in all bioinks in practice-oriented bioprinting, no significant difference in cell viability during the first 7 days of culture among four groups could be well explained. This may also suggest that the solvent concentration selected for bioprinting did not induce adverse cellular response and Alg-Gel hydrogel might require longer time to facilitate enhanced control over embedded cells.

Three-dimensional structural stability and cell compatibility were also considered an important prerequisite to obtain bioink printability. According to the present results, B-3 and B-4 were regarded less ideal for several reasons. Firstly, excessive high PBS ionic strength in B-3 and B-4 probably weakened the interaction (or connection) among macromolecules and prevent the crosslink of alginate scaffolds, which might cause the non-transparency, fast swelling and degradation of bioprinted constructs^25^. Secondly, insufficient cell spreading in B-3 and B-4 could be explained by SEM images on which Alg-Gel bioink was too loose to provide viable sites for cell spreading. This might be the reason why most cells stayed individual and aggregative cell clusters was seldom seen in B-3 and B-4. B-1 showed good printing resolution and property of observability and shape-maintenance. However, lower cell proliferation and aggregation in bioprinted constructs B-1 during culture *in vitro*, which were probably caused by its excessive high stiffness, limited its further application in extrusion-based cell-laden bioprinting.

By contrast, B-2 showed better properties and printability as well as best promotion of *in vitro* cell behaviors. The amounts of aggregative cell clusters increased from Day14 to Day28 of culture, indicating that the cells can generally form a glandular morphology inside the bioprinted constructs. It was also suggested that B-2 provide the suitable spacial environment for cell-cell interactions during ESCs differentiation into sweat gland lineage. Additionally, in most of the cell clusters in B-2 at Day14 of culture, K5, K14, K8 and K18 were all positive probably implied the cells of clusters were in different phases of differentiation. According to the hypothesis and explanation by Yang *et al*., it could be explained that some of ESCs differentiated immediately while others retained potency^26^. Although not directly explored in the present work, the effects of bioink properties on tissue formation has been studied extensively in the past, and the final sweat gland morphogenesis was probably a result of multi-factor interaction, such as growth factors^6^, biomaterial catogory^13^, printing methods^24^, composition of bioink^18^, and may indeed be found to act synergistically during tissue formation.

## Conclusion

Based on the test data, we demonstrated that printability and mechanical property of bioink, characters of printed constructs as well as the behaviors of embedded stem cells were influenced by solvent ionic strength of Alg-Gel hydrogel. Bioink B-2 was screened as an ideal candidate for facilitating ESCs proliferation and differentiation, indicating that this approach might help to optimize the 3D bioprinting application for sweat gland regeneration.

## Materials and Methods

### Isolation and Expansion of ESCs

The mouse ESCs were collected from dorsal skin of E12.5 embryonic mice [C57BL/6-Tg (ACTB-EGFP) 10 sb/J, Jackson Laboratory]. Briefly, E12.5 embryonic mice were executed and immersed in 75% ethanol (Beijing Chemical Works, Beijing, China), of which the dorsal skin was then separated and diced into 0.5 cm^2^ pieces and sterilized in 20 ml phosphate buffered saline (PBS, pH = 7.3) containing 10% penicillin-streptomycin for 20 minutes. Epidermis was detached from dermis by immersed in Dispase II (2 mg/ml, Sigma) for 30 minutes, after which it was immersed in collagenase I (2 mg/ml, Sigma) for another 30 minutes to remove the remaining dermis. Then, the epidermis was minced into 1 mm^2^ pieces and digested in 0.25% Trypsin-EDTA for 30 minutes. All processes above were performed in 37 °C. Finally, primary ESCs were harvested and incubated with medium [Nutrient Mixture F-12 (Ham) (1:1) D-MEM/F-12] (Gibco) supplemented with 10% fetal bovine serum (Gibco) and 1% penicillin-streptomycin (Gibco). Passage 2 to 4 of ESCs with density of 1 × 10^7^ cells/ml was prepared for the processes below. All animal experiments were carried out in accordance with the guidelines of the Institutional Animal Care and Use Committee of Chinese PLA General Hospital (Beijing, China). All experimental protocols were approved by the Institutional Animal Care and Use Committee of Chinese PLA General Hospital (Beijing, China).

### Preparation of Alg-Gel Bioink

The Alg-Gel bioink in each group was characterized by changing the ionic strength of PBS (Table 1). Bioink was prepared according to the same procedure as previously described^11^. Briefly, 10 ml bioink for each printing syringe was composed of 3 ml hyperthermia-dissolved Alg solution (8%, w/v), 6 ml Gel solution (20%, w/v) and 1 ml F12 medium containing 1 × 10^7^ ESCs (Passage 2 to 4) and 500 µL PD (plantar dermis homogenate prepared as previously described^6^) with sufficient mixing. In addition, the prepared solution of Alg and Gel were autoclaved before the mixing process which was strictly sterilized.

**Table 1:** Basic composition of Alg-Gel bioink (^a^F12 medium with cells; ^b^formula of solvent 1.0 × PBS: NaH_2_PO_4_ 8 mM, NaCl 136 mM, KH_2_PO_4_ 2 mM and KCl 2.6 mM. 0.5 × PBS owns half the concentration of 1.0 × PBS and 2.0 × PBS owns twice the concentration of 1.0 × PBS; ^c^distilled water).

Bioink	Concentration of Gelatin Solution	Concentration of Alginate Solution	Gel: Alg: Medi^a^ (volume)	Solvent^b^	PBS Ionic Strength
B-1	20% (w/v)	8% (w/v)	6:3:1	0 × PBS (DW^c^)	0 M
B-2	20% (w/v)	8% (w/v)	6:3:1	0.5 × PBS	0.082 M
B-3	20% (w/v)	8% (w/v)	6:3:1	1.0 × PBS	0.165 M
B-4	20% (w/v)	8% (w/v)	6:3:1	2.0 × PBS	0.328 M

### Rheological Test of Alg-Gel Bioink

Rheological test of Alg-Gel bioink was performed on the Haake RS6000 Rheometer with a plate sensor system^27^ (PP35-TiL, gap width = 0.1 mm and sample volume of 3.0 cm^3^). The temperature of the plate sensor system was maintained 10 °C (the working temperature of 3D bioprinter) throughout the measurement process by the liquid temperature-controlling unites of rheometer (Thermo).

The viscosity (Viscosity curve) and shear force (Shear Force Shear Rate curve) of bioink were measured by increasing the shear rate from 0 to 1500 sec^−1^ in 200 seconds and measure points were selected linearly.

The actual shear force acted on cells when they were extruded out of the printing nozzle was measured indirectly. Briefly, *T* (time of 2 ml bioink extrusion) was recorded and put into the Eq. (1) below:

1 $$\dot{\gamma }=8{V}/\pi {d}^{3}T$$

γ: shear rate (s^−1^); *V*: volume of Alg-Gel bioink (2 ml); *d*: diameter of printing nozzle (0.34 mm); *T*: time of extrusion (second). Then the actual shear force act on cells was determined according to the actual shear rate of each group. 3 samples were scored in 3 independent replicates for each group.

The dynamic frequency sweep (frequency range between 0.1 and 10.0 Hz) was conducted with 5% strain, in which storage modulus (G’) and loss modulus (G”) were measured.

### 3D Bioprinting Process with Alg-Gel Bioinks

Using our previously optimized printing process^11^, the printing was performed on the bioprinting platform (Regenovo 3D Bioprinter, China) with a liquid temperature controller (Thermo). Briefly, 10 ml Alg-Gel bioink was loaded into a pre-sterilized syringe. After a cooling process of storage at 0 °C for 30 minutes, the syringe loaded cooled bioink was equipped on the precooled print arm (10 °C) of the platform, while a sterilized 60 mm Petri dish as a substrate was temporal-fixed on the precooled print platform (10 °C). As the printing progressed, a 3D columnar construct (theoretically 40 mm diameter and 3 mm thickness) was formed by extruded continuous line of bioink, on which square pores (theoretically 2.5 mm width) were formed with layer-by-layer rotation of meandering thread pattern. Freshly printed construct was immediately crosslinked by immersing in 2 ml sterilized 10% calcium chloride solution for 10 min at 0 °C. Finally, the remnant calcium chloride solution was abandoned and the printed construct was cultured in the sweat gland cell (SGC) medium (200 ml DMEM and 200 ml F12 supplemented with 20 ml fetal calf serum [FCS] (Gibco), 10 ng/ml epidermal growth factor [EGF] (Sigma), 2 ng/ml liothyronine sodium (Gibco), 0.4 μg/ml hydrocortisone succinate (Gibco), 1 ml/100 ml insulin-transferrin-selenium [ITS] (Gibco), 1 ml/100mlpenicillin-streptomycin solution) in the incubator at 37 °C in a humidified atmosphere of 5% CO_2_.

### Characteristics of Bioprinted Constructs

The surface morphology was assessed by scanning electron microscope (JCM-6000 Versatile Benchtop SEM, JEOL), before which the 3D bioprinted constructs was freeze-dried (LGJ-12 vacuum freeze dryer, SongyuanHuaxing, Beijing) and sprayed (ETD-200 sputter coater for SEM, China).

Swelling ratio was detected by measuring the diameter of construct samples. Briefly, crosslinked bioprinted construct samples were incubated in F12 medium at 37 °C for 14 days. The initial diameter of bioprinted construct was *DMo*. And the diameter of construct, named *DMt*, was measured at predetermined time points (*t*). Swelling ratio of swollen construct sample was determined by Eq. (2). 3 samples were scored in 3 independent replicates for each group.

2 $${Swellingratio}({t})={(DMt/DMo)}^{3}\times {100} \% $$

Degradation rate of bioprinted construct was detected by quantifying the decrease of weight. Briefly, freshly bioprinted construct sample was weighed (*Wo*) and incubated in F12 medium at 37 °C for 14 days. At predetermined time points (*t*), bioprinted construct samples were dried and weighed (*Wt*). Degradation rate of construct sample was determined by Eq. (3). 3 samples were scored in 3 independent replicates for each group.

3 $$Degradationrate(t)=(Wo-Wt)/Wo\times {100} \% $$

### Cell Viability and Proliferation Analysis

To observe the time-varying viability of ESCs in bioprinted constructs, the Live/Dead assay kit [Eugene, Oregon, USA - 541. 445. 8300] was applied for cell staining to distinguish live cells (green fluorescence) from dead cells (red fluorescence) under fluorescent microscope (Olympus, BX51). Five individual randomized visual fields of one sample under microscope were chosen for live/dead calculation and three samples were analyzed for each group at each time point (D0, D1, D3, D7 and D14). 3 independent replicates were conducted in each group.

To detect proliferative activity of embedded ESCs, the bioprinted constructs were fixed in 4% paraformaldehyde at room temperature for 20 minutes and were stained with Ki-67 according to standard immunofluorescence protocols. Briefly, after fixation and antigen block, bioprinted constructs were incubated overnight at 4 °C with the Ki-67 primary antibody. Then bioprinted constructs were immersed in red fluorophore-labeled rabbit anti-mouse secondary antibody (1:300) for 2-hour dark incubation at room temperature. Finally, incubated constructs were mounted by DAPI Fluoromount-G (Southern Biotech, USA) and pictures were taken with a fluorescence microscope (Olympus, BX51) within 24 hours. At least 5 views of 3 samples were scored in 3 independent replicates for each group.

### Cell Aggregation and Differentiation in Bioprinted Constructs

To observe cell aggregation in the bioprinted constructs, pictures were taken continuously at D1, D3, D7, D14 and D28 of culture *in vitro* using an optical microscope system (Olympus) and a fluorescence microscope (Olympus, BX51). To detect differentiation of embedded ESCs, the bioprinted constructs were fixed in 4% paraformaldehyde at room temperature for 20 minutes and then were stained with tissue-specific markers according to standard immunofluorescence protocols. Briefly, after fixation and antigen block, bioprinted constructs were incubated overnight at 4 °C with the primary antibody below: the antibodies rabbit monoclonal anti-cytokeratin 5 (K5, 1:500, Abcam) and anti-cytokeratin 14 (K14, 1:500, Abcam); and SGC’s specific markers, the antibodies rabbit monoclonal anti-cytokeratin 8 (K8, 1:500, Abcam) and mouse monoclonal anti-cytokeratin 18 (K18, 1:500, Abcam). K5, K8 and K14 were immersed with red fluorophore-labeled mouse anti-rabbit secondary antibody (1:300) while K18 was immersed with red fluorophore-labeled rabbit anti-mouse secondary antibody (1:300) for 2-hour dark incubation at room temperature. Finally, incubated sections were mounted by DAPI Fluoromount-G (Southern Biotech, USA) and pictures were taken with a fluorescence microscope (Olympus, BX51) within 24 hours. At least 5 views of 3 samples were scored in 3 independent replicates for each group.

### Statistical analysis

All data presented are expressed as means ± standard deviations. Single-factor analysis of variance (ANOVA) combined with a Student Newman Keuls (SNK) post hoc test was used as the statistical test, and the significance level was set at 0.01 < *p* < 0.05 (^*^) and *p* < 0.01(^**^).
